# The healthcare value of the Magee Decision Algorithm™: use of Magee Equations™ and mitosis score to safely forgo molecular testing in breast cancer

**DOI:** 10.1038/s41379-020-0521-4

**Published:** 2020-03-17

**Authors:** Rohit Bhargava, Beth Z. Clark, Gloria J. Carter, Adam M. Brufsky, David J. Dabbs

**Affiliations:** 1 Departments of Pathology, Magee-Womens Hospital of UPMC, Pittsburgh, PA USA; 2 Departments of Medical Oncology, Magee-Womens Hospital of UPMC, Pittsburgh, PA USA; 3 Present Address: John A. Burns University of Hawaii Cancer Center, Honolulu, HI USA

**Keywords:** Breast cancer, Predictive markers

## Abstract

Magee Equations™ are multivariable models that can estimate onco*type* DX® Recurrence Score, and Magee Equation 3 has been shown to have chemopredictive value in the neoadjuvant setting as a standalone test. The current study tests the accuracy of Magee Decision Algorithm™ using a large in-house database. According to the algorithm, if all Magee Equation scores are <18, or 18–25 with a mitosis score of 1, then oncotype testing is not required as the actual oncotype recurrence score is expected to be ≤25 (labeled “do not send”). If all Magee Equation scores are 31 or higher, then also oncotype testing is not required as the actual score is expected to be >25 (also “do not send”). All other cases could be considered for testing (labeled “send”). Of the 2196 ER+, HER2-negative cases sent for oncotype testing, 1538 (70%) were classified as “do not send” and 658 (30%) as “send”. The classification accuracy in the “do not send” group was 95.1%. Of the 75 (4.9%) discordant cases (expected score ≤25 by decision algorithm but the actual oncotype score >25), 26 received endocrine therapy alone. None of these 26 patients experienced distant recurrence (average follow-up of 73 months). The Magee Decision Algorithm accurately identifies cases that will not benefit from oncotype testing. Such cases constitute ~70% of the routine clinical oncotype requests, an estimated saving of $300,000 per 100 test requests. The occasional discordant cases (expected ≤25, but actual oncotype score >25) appears to have an excellent outcome on endocrine therapy alone.

## Introduction

Several molecular tests are now regularly used in the management of breast cancer in routine clinical practice. Although developed mostly as prognostic assays, the majority of the testing is performed to make therapy decisions in hormone receptor-positive breast cancers. The most commonly used assay in the United States is onco*type* DX®. Based on earlier studies that utilized tissue blocks from National Surgical Adjuvant Breast and Bowel Project B-14 and B-20 clinical trials, oncotype clinical risk (of recurrence) categories were defined as low-risk (score 0 to <18, average risk of 7% assuming patient receives tamoxifen for 5 years), intermediate-risk (score 18–30, average risk 14%), and high-risk (score 31 or higher, average risk approaching 30%) [1, 2]. These retrospective studies showed the benefit of chemotherapy only in the high-risk group with no benefit in low risk and negligible benefit in intermediate-risk group [1, 2]. However, instead of using these predefined group scores, the prospective clinical trial (**T**rial **A**ssigning **I**ndividua**L**ized **O**ptions for **T**reatment or TAILORx) designed to assess the usefulness of oncotype testing redefined the intermediate-risk group as score 11–25. Consequently, patients with scores 0–10 received only endocrine therapy, patients with scores >25 received both endocrine and chemotherapy. Patients with oncotype recurrence score 11–25 were randomized to receive either endocrine therapy alone or both endocrine and chemotherapy. After 9 years of average follow-up, the recurrence rate and survival were similar between the endocrine only group and the chemo-endocrine group concluding that there is a lack of chemotherapy benefit in patients with recurrence score 11–25 [3]. Although practice changing, the results are not entirely unexpected. The earlier oncotype validation studies and a recent retrospective study of the Surveillance Epidemiology End Result database showed similar results [4].

We have previously designed multivariable models called Magee Equations™ (ME) that can estimate oncotype score [5, 6]. These models use routinely reported histopathology and breast cancer biomarker data to provide a score similar to oncotype. One of the equations (ME3) has been shown to predict for a pathologic complete response to neoadjuvant chemotherapy in ER+/HER2-negative tumors [7]. With the new oncotype recurrence score cut-off value of 25, we recently described a decision algorithm using MEs and tumor mitotic activity score to safely forgo oncotype testing [8].

The primary goal of the current study was to evaluate the accuracy of the Magee Decision Algorithm^TM^ within a large database. The secondary goal was to determine the clinical outcome for cases where the results are deemed discordant.

## Methods

**Fig. 1:**
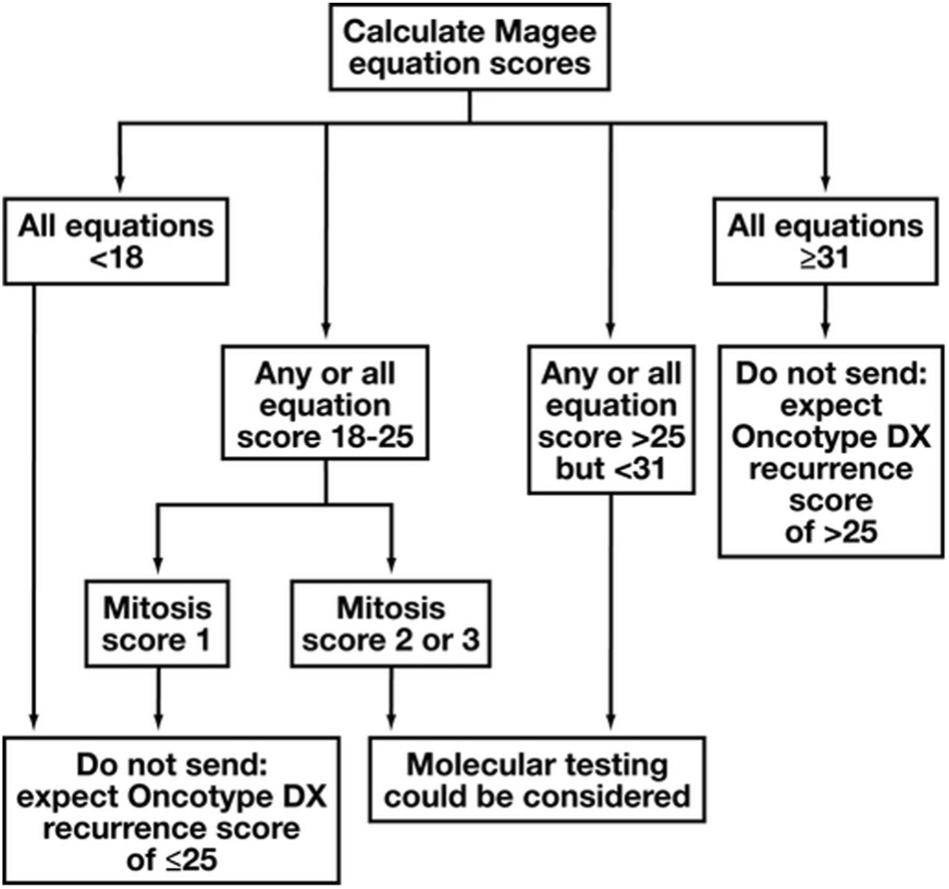
Magee Decision Algorithm™. Adapted from Bhargava et al. [8].

The current study tests the accuracy of the Magee Decision Algorithm™ using a large in-house database. According to the algorithm (Fig. 1), if all ME scores are <18, or 18–25 with a mitosis score of 1, then oncotype testing is not required as the actual oncotype recurrence score will be ≤25 (these cases were labeled as “do not send-expect low risk”). If all ME scores are 31 or higher, then also oncotype testing is not required as the actual score will be >25 (labeled as “do not send-expect high risk”). All other cases, i.e., any or all ME scores 18–25 and mitosis score >1, and any or all ME scores >25 to <31 regardless of the mitosis score could be considered for testing (labeled as “send’). The triage of cases as “send” or “do not send” was compared with actual oncotype recurrence score results. We analyzed all ER+, HER2-negative cases (including HER2 immunohistochemical score 2+ cases with HER2 copies of 4 to <6, such cases are classified as equivocal for ME score calculation) sent for oncotype testing with available pathology parameters for calculation of all MEs. The cases included in the study are from two in-house databases, a “retrospective” cohort (cases sent for clinical oncotype testing from 2007 to 2015; 1824 cases) and the cohort of cases used for “prospective” value study (cases sent for clinical oncotype testing in last 3 years; 372 cases), the partial results for which were recently published [8]. This resulted in a total of 2196 cases that formed the basis of this study.

Other details regarding variables required for calculation of ME scores are provided within Supplementary information (Supplementary data- methods).

For comparison of means, independent sample *t*-tests were performed. Univariable analysis was performed using *χ*^2^ and Fisher exact tests to compare the differences in percentages between groups. A *p* value < 0.05 was considered significant. Kaplan–Meier survival curves for distant recurrence free survival were analyzed for “discordant” cases (i.e., expected score ≤25 but actual oncotype score >25) and the *p* values were obtained using log-rank test (GraphPad Prism software, version 8.3.0, San Diego, CA).

## Results

The age of patients ranged from 26 to 87, with a median age of 59 years. Most were early-stage breast cancers. The median tumor size was 1.6 cm. Of the 2196 cases, 1879 (86%) were lymph node negative. The 2196 cases included 503 grade 1 (23%), 1352 grade 2 (61%), and 36 grade 3 (16%) tumors. A higher number of grade 2 tumors indicate the selection bias for requesting clinical oncotype testing. All cases were estrogen receptor (ER) positive and 2018 (92%) were progesterone receptor (PR) positive. All cases were HER2 negative, including the 53 or 2% cases with HER2 immunohistochemical score 2+ and HER2 copies of 4 to <6 per cell by fluorescence in situ hybridization.

**Fig. 2:**
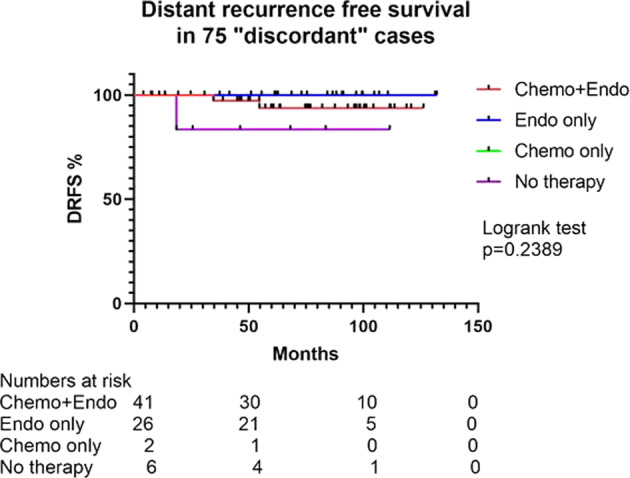
Distant recurrence free survival in 75 “discordant” cases. Distant recurrence free survival (DRFS) of 75 cases deemed discordant (i.e., expected score ≤25, but actual onco*type* DX® score >25).

Of the 2196 cases, 1538 (70.1%) were classified as “do not send” and 658 (29.9%) as “send”. The classification accuracy in the “do not send” group was 95.1% (see Table 1). Of the 75 discordant cases (expected ≤25, but actual oncotype >25, see Table 1), 41 received chemo-endocrine therapy, 2 received chemotherapy only, 26 had endocrine therapy alone (mostly an aromatase inhibitor), and 6 did not receive any systemic therapy (Fig. 2). The average follow-up was 71 months. The follow-up duration was similar for the chemo-endocrine therapy group (average: 71.9 months; interquartile range of 49.8–98.5 months) and in the endocrine therapy alone group (average: 72.9 months; interquartile range of 52.2–95.4 months). There were three distant recurrences, two in patients that received chemo-endocrine therapy and one in a patient who did not receive any systemic therapy. No distant recurrences were recorded in the group that received hormonal therapy alone. Two of the patients with recurrence died of disease (one patient who received chemo-endocrine therapy and one patient who did not receive any systemic therapy). There were two other deaths in the cohort but the cause was unrelated to breast cancer. The average age was 61 years for these 75 “discordant” cases with 9 patients being age 50 and below. Four of these young patients received chemo-endocrine therapy and four received endocrine therapy alone and one received no systemic therapy. As mentioned above, no recurrences and deaths were noted in patients who received endocrine therapy alone. The clinical–pathologic features of these 75 “discordant” cases were compared with 1443 “concordant” (expected ≤25 and actual oncotype ≤25) cases. The only parameter that showed statistically significant difference was PR expression (Table 2).

**Table 1:** Actual onco*type* DX® recurrence scores and Magee Equation scores on cases labeled as “do not send” (*n* = 1538).

	Do not send-expect high risk	Do not send-expect low risk	Total
Actual oncotype score >25	19	75	94
Actual oncotype score ≤25	1	1443	1444
Total	20	1518	1538

Excludes 658 cases labeled as “send”. Accuracy of “do not send”: 19  +  1443/1538 = 95.1%. Accuracy of “do not send-expect low” (≤25): 1443/1518 = 95.1%. Accuracy of “do not send-expect high” (>25): 19/20 = 95%.

**Table 2:** Clinical–pathologic characteristics of cases deemed “discordant” compared with “concordant” cases.

	Discordant (expected ≤ 25, actual > 25), *n* = 75	Concordant (expected ≤ 25, actual ≤ 25), *n* = 1443	*p* value
Age, mean	61 years	59 years	0.1352
Tumor size, mean	1.7 cm	1.8 cm	0.3404
Nottingham Grade			
Grade I	18 (24%)	474 (33%)	0.1289
Grade II	56 (75%)	966 (67%)	
Grade III	1 (1%)	3 (<1%)	
PR status			
Negative	20 (27%)	68 (5%)	<0.0001
Positive	55 (73%)	1375 (95%)	
PR H-score mean	96	178	<0.0001
ER H-score mean	260	262	0.5945
Ki-67 index mean	16	14	0.0585
Nodal status			
Negative	63 (84%)	1249 (87%)	0.8535
Positive	9 (12%)	171 (12%)	
ITC	1 (1%)	5 (<1%)	
Unknown	2 (3%)	18 (1%)	

*ER* estrogen receptor, *PR* progesterone receptor, *ITC* isolated tumor cell.

Of the 2196 total cases, 513 patients were age 50 or less. The results in this cohort were similar to the overall results. Of the 513 cases, 333 (65%) were classified as “do not send” based on the Magee Decision Algorithm and the classification accuracy of the “do not send” group was 97%. Within this “do not send” group, the percentage of cases with actual oncotype score <21 was 87% and the percentage of cases with actual oncotype score <16 was 59%.

With regards to the cases classified as “send” (*n* = 658; ~30% of the entire cohort), 191 cases (29%) had actual oncotype of >25. The clinical–pathologic data of the cases labeled as “send” was compared with the cases labeled “do not send-expect low” (Table 3). As expected, the cases labeled as “send” showed more aggressive histopathologic features.

**Table 3:** Clinical–pathologic characteristics of cases classified as “send” compared with cases labeled as “do not send-expect low”.

	Labeled “send”; *n* = 658	Labeled “do not send-expect low”; *n* = 1518	*p* value
Age, mean	58 years	59 years	0.1664
Tumor size, mean	2.2 cm	1.8 cm	<0.0001
Nottingham Grade			
Grade I	11 (2%)	492 (32.5%)	<0.0001
Grade II	329 (50%)	1022 (67%)	
Grade III	318 (48%)	4 (0.5%)	
PR status			
Negative	81 (12%)	88 (6%)	<0.0001
Positive	577 (88%)	1430 (94%)	
PR H-score mean	129	173	<0.0001
ER H-score mean	241	262	<0.0001
Ki-67 index mean	35%	14%	<0.0001
Nodal status			
Negative	550 (84%)	1312 (86%)	0.1207
Positive	94 (14%)	180 (12%)	
ITC	4 (0.5%)	6 (0.5%)	
Unknown	10 (1.5%)	20 (1.5%)	
Histologic type			
Ductal, NST	338 (51%)	715 (47%)	<0.0001
Lobular, classic	27 (4%)	140 (9%)	
Other	27 (4%)	54 (4%)	
Not recorded	266 (40%)	609 (40%)	

*ER* estrogen receptor, *PR* progesterone receptor, *ITC* isolated tumor cell, *NST* no special type.

In addition, we examined the results with respect to individual equations and the mean equation score within the Magee Decision Algorithm™ (Table 4). The detailed result tables are provided within Supplementary data (Supplementary data- entire dataset).

**Table 4:** Results using all Magee Equations compared with using individual equations and average equation score in the decision algorithm.

	All equations	ME1	ME2	ME3	Average
Percentage classified as “do not send”	70.1%	75.3%	74.3%	78.8%	75.8%
Percentage classified as “send”	29.9%	24.7%	25.7%	21.2%	24.2%
Classification accuracy of “do not send”	95.1%	93.9%	93.8%	94.2%	94.3%
Accuracy of “do not send-expect low”	95.1%	94.3%	94%	94.6%	94.3%
Accuracy of “do not send-expect high”	95%	82%	81.1%	77.5%	93.9%

ME1 requires tumor size, Nottingham score, estrogen receptor (ER), progesterone receptor (PR), HER2, Ki-67 immunohistochemical data. ME2 requires all the variables similar to ME1 except Ki-67. ME3 requires only ER, PR, HER2, and Ki-67 semi-quantitative results.

*ME* Magee Equation.

The data were also analyzed separately for the retrospective cohort and the cases from the prospective value study. The results were similar to the combined dataset and the details are provided in the Supplementary data (Supplementary data- cases from retrospective dataset and Supplementary data- cases from prospective value study).

## Discussion

In recent decades, breast medical oncologists in the United States have been trying to de-escalate the use of chemotherapy in ER+, HER2-negative early-stage breast cancer. This approach seems to be taking hold but there is still a lot of variability in chemotherapy use. However, it is important to understand why chemotherapy was overused in the first place. It appears that the National Institute of Health consensus statement in the year 2000 was partly responsible for chemotherapy overuse [9]. This statement basically recommended the use of chemotherapy in any breast cancer >1 cm (both lymph node-positive and lymph node negative). No consideration was given to tumor grade despite having ample data regarding breast cancer grade and prognosis at the time [10]. Subsequent use of breast cancer biomarkers in routine practice and molecular characterization of breast cancer confirmed different prognostic groups of ER+ breast cancers and heterogeneous benefit from cytotoxic chemotherapy [11, 12]. Non-pathologists have been critical of the subjective nature of breast cancer grading but the observed variability in grading among pathologists is no worse than categorization of tumors by current molecular assays. In a comparative study, all molecular assays categorized a comparable number of cases as low or high risk, but at the individual tumor level there was significant variability [13, 14]. Nevertheless, medical oncologists continue to use molecular assays for making therapy decisions. The chemotherapy recommendation can be different for the same patient depending on the molecular assay utilized to make such a recommendation [13]. Onco*type* DX® remains the most frequently used test in the United States for making breast cancer systemic therapy decisions. Initially described as a three-tiered test (low [0 to <18], intermediate [18–30], and high risk [>31]), the cut-offs are now changed to low risk (≤25) or high risk (>25) based on the results of TAILORx prospective clinical trial [3]. The recently published results from the TAILORx study showed similar survival of patients with oncotype scores of 11–25 receiving either chemo-endocrine therapy or endocrine therapy alone. There was some benefit in disease-free survival (but not in overall survival) with chemo-endocrine therapy in premenopausal patients with scores 16–25. However, this slight additional benefit of chemotherapy in premenopausal patients could have been due to ovarian suppression. It is to be noted that most patients in the TAILORx trial received tamoxifen alone as the endocrine therapy, while the Suppression of Ovarian Function Trial and the Tamoxifen and Exemestane Trial have shown that aromatase inhibitor with ovarian suppression is a superior form of endocrine therapy [15–17]. It is questionable how much additional benefit one can derive from chemotherapy after being treated with aromatase inhibitor and ovarian suppression. After the publication of these prospective trial results, it is generally accepted that postmenopausal patients with oncotype scores 25 or less do not require chemotherapy. These results suggest that if routine pathologic examination can confidently predict for oncotype score of 25 or less in early-stage breast cancer, then it can of be significant clinical value and provides extraordinary healthcare value for patients.

Our group has previously published multivariable models to estimate the oncotype score, first as proof of principle and later as a clinically useful tool to decide if a particular tumor needs oncotype testing [5, 6]. The models, now commonly known as MEs have been shown to be strongly chemopredictive in the neoadjuvant setting and also appear to have prognostic value [7]. In light of the TAILORx results we recently described an algorithmic approach to safely forgo oncotype testing. In the previously published prospective value study, cases with all MEs scores of <18 and cases with scores 18–25 but mitosis score of 1 almost always showed an actual oncotype score of 25 or less [8]. We also showed that in rare discordant cases, there are generally noninvasive tumor factors that appear to alter the actual score [8]. The current study is large-scale validation of this Magee Decision Algorithm™.

For the current study, we used ER+/HER2-negative cases sent for clinical oncotype testing and had Pathology data for calculation of all 3 ME scores. Using this large database of over 2000 cases, we unequivocally show the clinical usefulness of the Magee Decision Algorithm™. When cases are classified as “do not send (expect low)”, then the likelihood of the actual oncotype score coming back as >25 is <5%. Interestingly, even in those rare cases where results are deemed discordant (estimated ≤25, actual >25), chemotherapy use in such patients did not show any survival benefit compared with patients who received only endocrine therapy (Fig. 2). Comparison of clinical–pathologic features of the cases deemed “discordant” with the “concordant” cases (i.e., estimated ≤25 and actual also ≤25) showed only progesterone expression to be significantly different (Table 2). This is a well-known fact that oncotype score is inversely related to PR expression levels [18–22]. However, when PR expression is the only variable driving up the oncotype score (the “discordant” cases in the current study), then it may not affect patient outcome when they are treated with endocrine therapy alone. This underscores the importance of using a multivariable model, such as MEs over a single variable.

In addition to the decision algorithm that utilizes all three equations, we also analyzed the data using individual equations and the average MEs score (see Supplementary data). Using individual equations in the decision algorithm slightly increased the percentage of cases classified as “do not send”, but the accuracy of “do not send” algorithm also decreased slightly, particularly impacting the ability to predict “do not send-expect high risk” category (Table 4). The results for the use of the average ME score are almost similar to using all equations. Although results for each of the equations are comparable, the use of all equations slightly increases the accuracy of results and shall increase the user’s confidence to safely forgo oncotype testing.

**Fig. 3:**
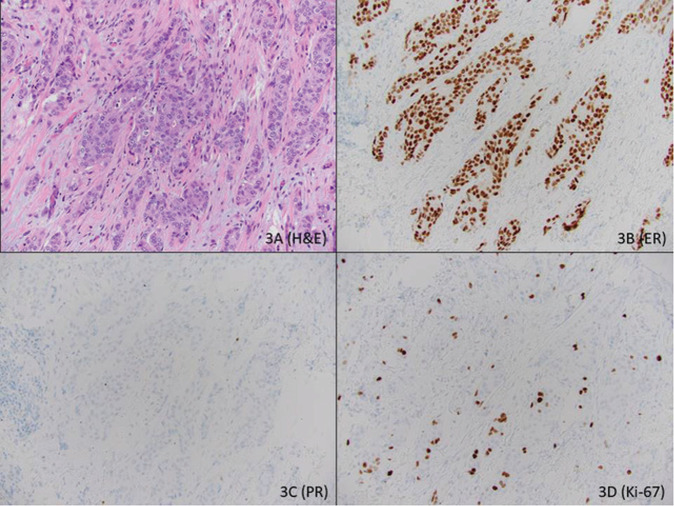
Example of a common type of ER positive breast cancer. Hematoxylin and eosin stained section of an invasive ductal carcinoma (**a**), grade II with Nottingham score of 6 (tubule formation score: 3; nuclear pleomorphism score: 2; mitotic activity score: 1). The tumor is diffusely and strongly positive for estrogen receptor with an H-score of 300 (**b**), but is negative for progesterone receptor with H-score of 0 (**c**). The tumor is negative for HER2 (not shown) with a Ki-67 labeling index of 15% (**d**).

Our group was the first to suggest that routine histopathologic data can estimate the oncotype score and also defined a multivariable model in 2008 which was revised in 2013 [5, 6]. Since then, there have been several publications that have either validated MEs or defined other similar models [18, 19, 21–39]. However, the accuracy and simplicity of MEs make it easier to use in routine practice to make confident clinical decisions. MEs require and also provide more granular data to make therapy decisions compared with other published models. This can be explained by taking a hypothetical example comparing MEs with the University of Tennessee Medical Center (UTMC) Nomogram which was recently updated after the TAILORx trial results [33, 34]. The example is of a common type of ER+ breast cancer, i.e., a 55-year-old patient with 2.0 cm, grade II (Nottingham score 6, with mitosis score of 1), lymph node negative, ER+ (H-score of 300), PR negative (H-score 0), HER2 negative, Ki-67 labeling index of 15% invasive ductal carcinoma (Fig. 3). Using the University of Tennessee Nomogram (https://utgsm.shinyapps.io/OncotypeDXCalculator/), the probability of a low-risk oncotype is 53% and the probability of a high-risk oncotype is 47%. However, the estimated ME scores (https://path.upmc.edu/onlineTools/mageeequations.html) on this case are 21.5 (equation 1), 21.7 (equation 2), and 20.6 (equation 3). Using the Magee Decision Algorithm (equation results between 18 and 25 and mitosis score of 1), this case will result in an actual oncotype score of 25 or less with over 95% certainty. In such cases, one could forgo oncotype testing using Magee Decision Algorithm but this decision cannot be taken based on UTMC Nomogram results.

Our study has enormous cost-saving implications. The cases included in this study are the cases sent for clinical oncotype testing, mostly requested by breast medical oncologists. Medical oncologists at our institution generally follow guidelines set forth by national societies (American Society of Clinical Oncology and National Comprehensive Cancer Network), but there is individual variation in ordering oncotype based on individual patient factors. There was no bias in case selection except that there was preponderance of Grade II cases, which are considered “borderline” for treatment purposes. Based on Magee Decision Algorithm™, the oncotype testing could have been avoided in 70% of the cases without having any negative clinical impact. Magee Decision Algorithm utilizes morpho-immunohistologic variables from a routine pathology report for which there is no additional cost. The calculator for MEs is available online on the department website for anyone to use for free (https://path.upmc.edu/onlineTools/mageeequations.html). Even login information is not required. In contrast, oncotype testing costs over $4000 per test. For every 100 tests, the institution/insurance could have saved ~$300,000 without impacting patient care. The counter-argument that savings from avoiding chemotherapy based on oncotype far outweighs the cost of the assay is not valid as similar savings can be attained using MEs/Magee Decision Algorithm. Additional savings come from safely forgoing oncotype testing. Others have also reported significant cost savings with the use of MEs [37, 38, 40]. This should alarm integrated health systems (provider and insurer) that want to move toward value-based system [41].

The study strength is that it utilized a large database to test the validity of the decision algorithm. In addition, the pathology slides were not reviewed for this study and the results from the report were taken as-is for calculation of ME scores. This is what is expected in routine practice. There has been some concern regarding MEs or similar models that require semi-quantitative results with respect to standardization and reproducibility [42, 43]. However, we have shown good interobserver concordance for H-scores [44]. For Ki-67 evaluation, the pathologists at our institution have often used a more pragmatic approach rather than actual counting of 500 or 1000 tumor cells [45, 46]. We first estimate the Ki-67 labeling index. If the estimate falls below 10 or above 50, then estimate stands as the final Ki-67 labeling index. If the estimate is between 10 and 50, then 50–100 cells are counted in a representative area based on the pathologist’s discretion to arrive at the labeling index. This approach seems to have worked as seen in this study and our prior neoadjuvant study, where the Ki-67 labeling index has been used in a multivariable model to predict chemotherapy benefit [7]. One potential weakness of the study is that all cases are from one institution where pathology reports are signed out by breast pathologists. There are published studies on the usefulness of MEs from other institutions, but it is unclear how the Magee Decision Algorithm™ will perform at other academic and nonacademic institutions. This study can be used as a springboard for studies at other institutions or a multi-institutional study.

After years of criticism regarding tumor grading and subjective reporting by pathologists, this study clearly shows the value of semi-quantitative scoring and using pathology-derived information in a cohesive manner that clinicians can understand. The data presented in this study provide a strong argument in favor of including MEs™ for stratifying patients in clinical trials. Magee Decision Algorithm™ provides an effective method to safely forgo onco*type* DX® testing. This approach will save both time and valuable resources. This is particularly valuable for large institutions and/or integrated health systems.

## Supplementary information

Supplemental material

## References

[1] Paik S, Shak S, Tang G, Kim C, Baker J, Cronin M (2004). A multigene assay to predict recurrence of tamoxifen-treated, node-negative breast cancer. N Engl J Med.

[2] Paik S, Tang G, Shak S, Kim C, Baker J, Kim W (2006). Gene expression and benefit of chemotherapy in women with node-negative, estrogen receptor-positive breast cancer. J Clin Oncol.

[3] Sparano JA, Gray RJ, Makower DF, Pritchard KI, Albain KS, Hayes DF (2018). Adjuvant Chemotherapy guided by a 21-gene expression assay in breast cancer. N Engl J Med.

[4] Petkov VI, Miller DP, Howlader N, Gliner N, Howe W, Schussler N (2016). Breast-cancer-specific mortality in patients treated based on the 21-gene assay: a SEER population-based study. NPJ Breast Cancer.

[5] Flanagan MB, Dabbs DJ, Brufsky AM, Beriwal S, Bhargava R (2008). Histopathologic variables predict oncotype DX recurrence score. Mod Pathol.

[6] Klein ME, Dabbs DJ, Shuai Y, Brufsky AM, Jankowitz R, Puhalla SL (2013). Prediction of the oncotype DX recurrence score: use of pathology-generated equations derived by linear regression analysis. Mod Pathol.

[7] Farrugia DJ, Landmann A, Zhu L, Diego EJ, Johnson RR, Bonaventura M (2017). Magee Equation 3 predicts pathologic response to neoadjuvant systemic chemotherapy in estrogen receptor positive, HER2 negative/equivocal breast tumors. Mod Pathol.

[8] Bhargava R, Clark BZ, Dabbs DJ (2019). Breast cancers with Magee Equation Score of less than 18, or 18-25 and Mitosis Score of 1, do not require oncotype DX testing: a value study. Am J Clin Pathol.

[9] Eifel P, Axelson JA, Costa J, Crowley J, Curran WJ, Deshler A, et al. National Institutes of Health Consensus Development Conference statement: adjuvant therapy for breast cancer, November 1–3, 2000, J Natl Cancer Inst Monogr. 2001;5–15. 10.1093/jnci/93.13.979 11438563

[10] Elston CW, Ellis IO (1991). Pathological prognostic factors in breast cancer. I. The value of histological grade in breast cancer: experience from a large study with long-term follow-up. Histopathology.

[11] Sorlie T, Perou CM, Tibshirani R, Aas T, Geisler S, Johnsen H (2001). Gene expression patterns of breast carcinomas distinguish tumor subclasses with clinical implications. Proc Natl Acad Sci USA.

[12] Sorlie T, Tibshirani R, Parker J, Hastie T, Marron JS, Nobel A (2003). Repeated observation of breast tumor subtypes in independent gene expression data sets. Proc Natl Acad Sci USA.

[13] Bartlett JM, Bayani J, Marshall A, Dunn JA, Campbell A, Cunningham C (2016). Comparing breast cancer multiparameter tests in the OPTIMA prelim trial: no test is more equal than the others. J Natl Cancer Inst.

[14] Stein RC, Dunn JA, Bartlett JM, Campbell AF, Marshall A, Hall P (2016). OPTIMA prelim: a randomised feasibility study of personalised care in the treatment of women with early breast cancer. Health Technol Assess.

[15] Francis PA, Pagani O, Fleming GF, Walley BA, Colleoni M, Lang I (2018). Tailoring adjuvant endocrine therapy for premenopausal breast cancer. N Engl J Med.

[16] Francis PA, Regan MM, Fleming GF, Lang I, Ciruelos E, Bellet M (2015). Adjuvant ovarian suppression in premenopausal breast cancer. N Engl J Med.

[17] Sparano JA, Gray RJ, Ravdin PM, Makower DF, Pritchard KI, Albain KS (2019). Clinical and genomic risk to guide the use of adjuvant therapy for breast cancer. N Engl J Med.

[18] Allison KH, Kandalaft PL, Sitlani CM, Dintzis SM, Gown AM (2012). Routine pathologic parameters can predict oncotype DX recurrence scores in subsets of ER positive patients: who does not always need testing?. Breast Cancer Res Treat.

[19] Auerbach J, Kim M, Fineberg S (2010). Can features evaluated in the routine pathologic assessment of lymph node-negative estrogen receptor-positive stage I or II invasive breast cancer be used to predict the Oncotype DX recurrence score?. Arch Pathol Lab Med.

[20] Clark BZ, Dabbs DJ, Cooper KL, Bhargava R (2013). Impact of progesterone receptor semiquantitative immunohistochemical result on Oncotype DX recurrence score: a quality assurance study of 1074 cases. Appl Immunohistochem Mol Morphol.

[21] Eaton AA, Pesce CE, Murphy JO, Stempel MM, Patil SM, Brogi E (2017). Estimating the OncotypeDX score: validation of an inexpensive estimation tool. Breast Cancer Res Treat.

[22] Gage MM, Rosman M, Mylander WC, Giblin E, Kim HS, Cope L (2015). A validated model for identifying patients unlikely to benefit from the 21-Gene Recurrence Score Assay. Clin Breast Cancer.

[23] Robertson SJ, Ibrahim MFK, Stober C, Hilton J, Kos Z, Mazzarello S (2019). Does integration of Magee Equations into routine clinical practice affect whether oncologists order the Oncotype DX test? A prospective randomized trial. J Eval Clin Pr.

[24] Robertson SJ, Pond GR, Hilton J, Petkiewicz SL, Ayroud Y, Kos Z (2020). Selecting patients for Oncotype DX testing using standard clinicopathologic information. Clin Breast Cancer.

[25] Chen YY, Tseng LM, Yang CF, Lien PJ, Hsu CY (2016). Adjust cut-off values of immunohistochemistry models to predict risk of distant recurrence in invasive breast carcinoma patients. J Chin Med Assoc.

[26] Geradts J, Bean SM, Bentley RC, Barry WT (2010). The oncotype DX recurrence score is correlated with a composite index including routinely reported pathobiologic features. Cancer Invest.

[27] Harowicz MR, Robinson TJ, Dinan MA, Saha A, Marks JR, Marcom PK (2017). Algorithms for prediction of the Oncotype DX recurrence score using clinicopathologic data: a review and comparison using an independent dataset. Breast Cancer Res Treat.

[28] Hou Y, Tozbikian G, Zynger DL, Li Z (2017). Using the modified Magee Equation to identify patients unlikely to benefit from the 21-Gene Recurrence Score Assay (Oncotype DX Assay). Am J Clin Pathol.

[29] Hou Y, Zynger DL, Li X, Li Z (2017). Comparison of Oncotype DX with modified Magee Equation recurrence scores in low-grade invasive carcinoma of breast. Am J Clin Pathol.

[30] Ingoldsby H, Webber M, Wall D, Scarrott C, Newell J, Callagy G (2013). Prediction of Oncotype DX and TAILORx risk categories using histopathological and immunohistochemical markers by classification and regression tree (CART) analysis. Breast.

[31] Khoury T, Huang X, Chen X, Wang D, Liu S, Opyrchal M (2016). Comprehensive histologic scoring to maximize the predictability of pathology-generated equation of breast cancer oncotype DX recurrence score. Appl Immunohistochem Mol Morphol.

[32] Kim HS, Umbricht CB, Illei PB, Cimino-Mathews A, Cho S, Chowdhury N (2016). Optimizing the use of gene expression profiling in early-stage breast cancer. J Clin Oncol.

[33] Orucevic A, Bell JL, King M, McNabb AP, Heidel RE (2019). Nomogram update based on TAILORx clinical trial results – Oncotype DX breast cancer recurrence score can be predicted using clinicopathologic data. Breast.

[34] Orucevic A, Bell JL, McNabb AP, Heidel RE (2017). Oncotype DX breast cancer recurrence score can be predicted with a novel nomogram using clinicopathologic data. Breast Cancer Res Treat.

[35] Sughayer M, Alaaraj R, Alsughayer A (2018). Applying new Magee Equations for predicting the Oncotype Dx recurrence score. Breast Cancer.

[36] Tang P, Wang J, Hicks DG, Wang X, Schiffhauer L, McMahon L (2010). A lower Allred score for progesterone receptor is strongly associated with a higher recurrence score of 21-gene assay in breast cancer. Cancer Invest.

[37] Turner BM, Gimenez-Sanders MA, Soukiazian A, Breaux AC, Skinner K, Shayne M (2019). Risk stratification of ER-positive breast cancer patients: a multi-institutional validation and outcome study of the Rochester Modified Magee algorithm (RoMMa) and prediction of an Oncotype DX((R)) recurrence score <26. Cancer Med.

[38] Turner BM, Skinner KA, Tang P, Jackson MC, Soukiazian N, Shayne M (2015). Use of modified Magee Equations and histologic criteria to predict the Oncotype DX recurrence score. Mod Pathol.

[39] Walts AE, Mirocha JM, Bose S (2018). Comparison of Magee and Oncotype DX Recurrence Scores in estrogen receptor positive breast cancers. Breast J.

[40] de Lima MAG, Clemons M, Van Katwyk S, Stober C, Robertson SJ, Vandermeer L, et al. Cost analysis of using Magee scores as a surrogate of Oncotype DX for adjuvant treatment decisions in women with early breast cancer. J Eval Clin Pract. 2019. https://www.ncbi.nlm.nih.gov/pubmed/31287198. [Epub ahead of print]. 10.1111/jep.13223 31287198

[41] Dabbs DJ, Clark BZ, Serdy K, Onisko A, Brufsky AM, Smalley S (2018). Pathologist’s health-care value in the triage of Oncotype DX((R)) testing: a value-based pathology study of tumour biology with outcomes. Histopathology.

[42] Gyorffy B, Hatzis C, Sanft T, Hofstatter E, Aktas B, Pusztai L (2015). Multigene prognostic tests in breast cancer: past, present, future. Breast Cancer Res.

[43] Vieira AF, Schmitt F (2018). An update on breast cancer multigene prognostic tests-emergent clinical biomarkers. Front Med.

[44] Cohen DA, Dabbs DJ, Cooper KL, Amin M, Jones TE, Jones MW (2012). Interobserver agreement among pathologists for semiquantitative hormone receptor scoring in breast carcinoma. Am J Clin Pathol.

[45] Dowsett M, Nielsen TO, A’Hern R, Bartlett J, Coombes RC, Cuzick J (2011). Assessment of Ki67 in breast cancer: recommendations from the International Ki67 in Breast Cancer working group. J Natl Cancer Inst.

[46] Polley MY, Leung SC, McShane LM, Gao D, Hugh JC, Mastropasqua MG (2013). An international Ki67 reproducibility study. J Natl Cancer Inst.

