# Supplementary material for: The healthcare value of the Magee Decision Algorithm™: use of Magee Equations™ and mitosis score to safely forgo molecular testing in breast cancer
Source: Mod Pathol. 2020 Mar 17;33(8):1563–70. doi: 10.1038/s41379-020-0521-4 (PMC7384988; doi:10.1038/s41379-020-0521-4)
Supplement: Supplementary file 1 — Supplemental material [file 41379_2020_521_MOESM1_ESM.pdf]

Table of contents

Pages 2-3: Methods

Pages 4-8: Entire dataset

Pages 9-13: Cases from retrospective dataset

Pages 14-18: Cases from prospective value study

The cases included in this study are clinical cases on which onco*type* DX® testing was requested by treating physicians. The data on tumor size, tumor grading and receptor studies were taken from Pathology reports for calculation of Magee Equation scores.

NOTTINGHAM SCORE: Breast tumor grading is performed using Nottingham grading system. The grading has 3 components; the tubule formation score, nuclear pleomorphism score, and mitotic activity score. Each component is scored from 1-3. Once individual scores for each component are assigned, the scores are added to get the Nottingham score that range from 3-9. The tumors with scores 3, 4, and 5 are grouped into grade I tumors, scores of 6 and 7 are grouped into grade II tumors, and the tumors with scores 8 and 9 are grade III tumors. Mitotic activity score is dependent on number of mitotic figures per 10 high power fields. Since the optical diameter of a high power field may vary between different scopes, Pathologists assign the mitotic activity scores based on field diameters. For a scope with field diameter of 0.55 mm, 0-8 mitotic figures per 10 high power field, the tumor gets a score of 1; for 9-18 mitotic figures per 10 high power fields, the tumor is assigned a score of 2; for >18 mitotic figures per 10 high power fields, the tumor is assigned a score of 3.

ESTROGEN/PROGESTERONE RECEPTORS (ER/PR) TEST DETAILS: The immunohistochemical staining was performed on clinical cases (mostly core biopsies) fixed in 10% neutral buffered formalin (8-72 hours) with appropriate positive and negative internal/external controls according to the 2010 American Society of Clinical Oncology-College of American Pathologist (ASCO-CAP) Guidelines. The case was classified as positive for Estrogen or Progesterone receptor when nuclear immunostaining is identified in greater than or equal to 1% of the tumor cells (i.e. and H-score of 1 or higher). The ER and PR Histologic Score (H-Score, or HS) is calculated as the sum of intensity of staining times the proportion of cells staining and has a dynamic range of 0 to 300.
Estrogen receptor antibody SP1, FDA approved, was performed using the IVIEW detection on the Benchmark ULTRA (Ventana, Tucson, AZ). Progesterone receptor antibody 1E2, FDA approved, was performed using the IVIEW detection on the Benchmark ULTRA (Ventana, Tucson, AZ).

HER2 IMMUNOHISTOCHEMISTRY TEST DETAILS: The immunohistochemical staining was performed on clinical cases (mostly core biopsies) fixed in 10% neutral buffered formalin (8-72 hours) with appropriate positive and negative external controls. The antibody clone, 4B5 was used as part of FDA approved Pathway on the Benchmark ULTRA (Ventana, Tucson, AZ) and interpreted according to the ASCO/CAP HER2 guidelines as follows: Score 0 (negative) = No staining is observed or incomplete faint/barely perceptible membranous staining is observed in ≤10% of the tumor cells. Score 1+ (negative) = A faint/barely perceptible membrane staining is detected in more than 10% of the tumor cells. The cells are only stained in part of their membrane. Score 2+ (equivocal) = Circumferential weak to moderate membrane staining is observed in more than 10% of the tumor cells. Intense circumferential membrane staining in ≤10% of tumor cells is also interpreted as score of 2+. This score required reflex testing by fluorescence in-situ hybridization (FISH). Score 3+ (positive) = A strong complete membrane staining is observed in more than 10% of the tumor cells.

Ki-67 IMMUNOHISTOCHEMISTRY TEST DETAILS: The immunohistochemical staining was performed on clinical cases (mostly core biopsies) fixed in 10% neutral buffered formalin (8-72

hours) with appropriate controls. Ki-67 labeling index was calculated as percent positive tumor cells in the entire tumor section. The test was performed using antibody clone 30-9 (Ventana).

<u>HER2 STATUS CLASSIFICATION (for Magee Equation score calculation):</u> Although HER2 cases are currently classified according to the ASCO/CAP 2018 update at our institution, a slightly modified approach is taken for Magee Equation score calculation. It is to be noted that the 2007 ASCO/CAP HER2 guideline criteria were utilized for classification of cases for initial development and validation of Magee Equations. However, the ASCO/CAP HER2 classification criteria have changed over the years. In the neoadjuvant study assessing the chemopredictiveness of Magee Equation 3 (Mod Pathol. 2017;30:1078-1085. PMID: 28548119), following criteria were used for determination of HER2 status:

HER2 negative: HER2 IHC score 0, IHC score 1+, and IHC score 2+ with HER2 copies per cell less than 4 by FISH

HER2 positive: HER2 IHC score of 3+, and HER2 IHC score of 2+ with HER2 copies per cell of 6 or more by FISH

HER2 equivocal: HER2 IHC score of 2+ with HER2 copies per cell of 4 to less than 6 by FISH

We have used the same criteria for HER2 status classification in the current study. Only HER2 negative and HER2 “equivocal” cases were included in the current study.

Entire dataset
ER+/HER2-neg/eq cases with all 3 Magee Equation scores: 2196
Based on all 3 equations (Magee Decision Algorithm):

**Oncotype Score**	**Do not send-expect high**	**Do not send-expect low**	**Send**	**Grand Total**
>25	19	75	191	285
25 or less	1	1443	467	1911
**Grand Total**	**20**	**1518**	**658**	**2196**

%age classified as do not send: 20+1518/2196 = 1538/2196=70%
%age classified as send: 658/2196= 30%

**Oncotype Score**	**Do not send-expect high**	**Do not send-expect low**	**Grand Total**
>25	19	75	94
25 or less	1	1443	1444
**Grand Total**	**20**	**1518**	**1538**

Accuracy of do not send: 19+1443/1538 = 95.1%
Ability to predict low-risk (≤25): 1443/1518= 95.1%
Ability to predict high-risk (>25): 19/20= 95%

Based on ME1 ONLY:

Oncotype Score	Do not send-expect high	Do not send-expect low	Send	Grand Total
>25	41	92	152	285
25 or less	9	1512	390	1911
**Grand Total**	**50**	**1604**	**542**	**2196**

%age classified as do not send: 50+1604/2196=75.3%
%age classified as send: 542/2196=24.7%

Oncotype Score	Do not send-expect high	Do not send-expect low	Grand Total
>25	41	92	133
25 or less	9	1512	1521
**Grand Total**	**50**	**1604**	**1654**

Accuracy of do not send: 41+1512/1654=93.9%
Accuracy of do not send (expect low): 1512/1604=94.3%
Accuracy of do not send (expect high): 41/50=82%

Based on ME2 ONLY:

**Oncotype Score**	**Do not send-expect high**	**Do not send-expect low**	**Send**	**Grand Total**
>25	30	95	160	285
25 or less	7	1500	404	1911
**Grand Total**	**37**	**1595**	**564**	**2196**

%age classified as do not send: 37+1595/2196=74.3%
%age classified as send: 564/2196=25.7%

**Oncotype Score**	**Do not send-expect high**	**Do not send-expect low**	**Grand Total**
>25	30	95	125
25 or less	7	1500	1507
**Grand Total**	**37**	**1595**	**1632**

Accuracy of do not send: 30+1500/1632=93.8%
Accuracy of do not send (expect low): 1500/1595=94%
Accuracy of do not send (expect high): 30/37=81.1%

Based on ME3 ONLY:

**Oncotype Score**	**Do not send-expect high**	**Do not send-expect low**	**Send**	**Grand Total**
>25	31	92	162	285
25 or less	9	1599	303	1911
**Grand Total**	**40**	**1691**	**465**	**2196**

%age classified as do not send: 40+1691/2196=78.8%
%age classified as send: 465/2196=21.2%

**Oncotype Score**	**Do not send-expect high**	**Do not send-expect low**	**Grand Total**
>25	31	92	123
25 or less	9	1599	1608
**Grand Total**	**40**	**1691**	**1731**

Accuracy of do not send: 31+1599/1731=94.2%
Accuracy of do not send (expect low): 1599/1691=94.6%
Accuracy of do not send (expect high): 31/40=77.5%

Based on ME-mean:

**Oncotype Score**	**Do not send-expect high**	**Do not send-expect low**	**Send**	**Grand Total**
>25	31	93	161	285
25 or less	2	1538	371	1911
**Grand Total**	**33**	**1631**	**532**	**2196**

%age classified as do not send: 33+1631/2196=75.8%
%age classified as send: 532/2196=24.2%

**Oncotype Score**	**Do not send-expect high**	**Do not send-expect low**	**Grand Total**
>25	31	93	124
25 or less	2	1538	1540
**Grand Total**	**33**	**1631**	**1664**

Accuracy of do not send: 31+1538/1664=94.3%
Accuracy of do not send (expect low): 1538/1631=94.3%
Accuracy of do not send (expect high): 31/33=93.9%

Cases from retrospective dataset

ER+/HER2-neg/eq cases with all 3 Magee Equation scores: 1824

Based on all 3 equations (Magee Algorithm):

**Oncotype Score**	**Do not send-expect high**	**Do not send-expect low**	**Send**	**Grand Total**
>25	16	67	166	249
25 or less	1	1182	392	1575
**Grand Total**	**17**	**1249**	**558**	**1824**

%age classified as do not send: 17+1249/1824 = 1266/1824=69.4%

%age classified as send: 558/1824= 30.6%

**Oncotype Score**	**Do not send-expect high**	**Do not send-expect low**	**Grand Total**
>25	16	67	83
25 or less	1	1182	1183
**Grand Total**	**17**	**1249**	**1266**

Accuracy of do not send: 16+1182/1266 = 94.6%

Accuracy of do not send (expect low): 1182/1249= 94.6%

Accuracy of do not send (expect high): 16/17= 94.1%

Based on ME1 ONLY:

**Oncotype Score**	**Do not send-expect high**	**Do not send-expect low**	**Send**	**Grand Total**
>25	35	81	133	249
25 or less	7	1240	328	1575
**Grand Total**	**42**	**1321**	**461**	**1824**

%age classified as do not send: 42+1321/1824=74.7%

%age classified as send: 461/1824=25.3%

**Oncotype Score**	**Do not send-expect high**	**Do not send-expect low**	**Grand Total**
>25	35	81	116
25 or less	7	1240	1247
**Grand Total**	**42**	**1321**	**1363**

Accuracy of do not send: 35+1240/1363=93.5%

Accuracy of do not send (expect low): 1240/1321=93.9%

Accuracy of do not send (expect high): 35/42=83.3%

Based on ME2 ONLY:

**Oncotype Score**	**Do not send-expect high**	**Do not send-expect low**	**Send**	**Grand Total**
>25	26	86	137	249
25 or less	5	1234	336	1575
**Grand Total**	**31**	**1320**	**473**	**1824**

%age classified as do not send: 31+1320/1824=74.1%
%age classified as send: 473/1824=25.9%

**Oncotype Score**	**Do not send-expect high**	**Do not send-expect low**	**Grand Total**
>25	26	86	112
25 or less	5	1234	1239
**Grand Total**	**31**	**1320**	**1351**

Accuracy of do not send: 26+1234/1351=93.3%
Accuracy of do not send (expect low): 1234/1320=93.5%
Accuracy of do not send (expect high): 26/31=83.8%

Based on ME3 ONLY:

**Oncotype Score**	**Do not send-expect high**	**Do not send-expect low**	**Send**	**Grand Total**
>25	26	81	142	249
25 or less	8	1313	254	1575
**Grand Total**	**34**	**1394**	**396**	**1824**

%age classified as do not send: 34+1394/1824=78.3%
%age classified as send: 396/1824=21.7%

**Oncotype Score**	**Do not send-expect high**	**Do not send-expect low**	**Grand Total**
>25	26	81	107
25 or less	8	1313	1321
**Grand Total**	**34**	**1394**	**1428**

Accuracy of do not send: 26+1313/1428=93.7%
Accuracy of do not send (expect low): 1313/1394=94.2%
Accuracy of do not send (expect high): 26/34=76.5%

Based on ME-mean:

**Oncotype Score**	**Do not send-expect high**	**Do not send-expect low**	**Send**	**Grand Total**
>25	26	84	139	249
25 or less	2	1264	309	1575
**Grand Total**	**28**	**1348**	**448**	**1824**

%age classified as do not send: 28+1348/1824=75.4%
%age classified as send: 448/1824=24.6%

**Oncotype Score**	**Do not send-expect high**	**Do not send-expect low**	**Grand Total**
>25	26	84	110
25 or less	2	1264	1266
**Grand Total**	**28**	**1348**	**1376**

Accuracy of do not send: 26+1264/1376=93.8%
Accuracy of do not send (expect low): 1264/1348=93.8%
Accuracy of do not send (expect high): 26/28=92.9%

Cases from prospective value study

ER+/HER2-neg/eq cases with all 3 Magee Equation scores: 372

Based on all 3 equations (Magee Algorithm):

**Oncotype Score**	**Do not send-expect high**	**Do not send-expect low**	**Send**	**Grand Total**
>25	3	8	25	36
25 or less		261	75	336
**Grand Total**	**3**	**269**	**100**	**372**

%age classified as do not send: 3+269/372 = 272/372=73.1%

%age classified as send: 100/372= 26.9%

**Oncotype Score**	**Do not send-expect high**	**Do not send-expect low**	**Grand Total**
>25	3	8	11
25 or less		261	261
**Grand Total**	**3**	**269**	**272**

Accuracy of do not send: 3+261/272 = 97.1%

Accuracy of do not send (expect low): 261/269= 97%

Accuracy of do not send (expect high): 3/3= 100%

Based on ME1 ONLY:

**Oncotype Score**	**Do not send-expect high**	**Do not send-expect low**	**Send**	**Grand Total**
>25	6	11	19	36
25 or less	2	272	62	336
**Grand Total**	**8**	**283**	**81**	**372**

%age classified as do not send: 8+283/372=78.3%
%age classified as send: 81/372=21.7%

**Oncotype Score**	**Do not send-expect high**	**Do not send-expect low**	**Grand Total**
>25	6	11	17
25 or less	2	272	274
**Grand Total**	**8**	**283**	**291**

Accuracy of do not send: 6+272/291=95.5%
Accuracy of do not send (expect low): 272/283=96.1%
Accuracy of do not send (expect high): 6/8=75%

Based on ME2 ONLY:

**Oncotype Score**	**Do not send-expect high**	**Do not send-expect low**	**Send**	**Grand Total**
>25	4	9	23	36
25 or less	2	266	68	336
**Grand Total**	**6**	**275**	**91**	**372**

%age classified as do not send: 6+275/372=75.5%
%age classified as send: 91/372=24.5%

**Oncotype Score**	**Do not send-expect high**	**Do not send-expect low**	**Grand Total**
>25	4	9	13
25 or less	2	266	268
**Grand Total**	**6**	**275**	**281**

Accuracy of do not send: 4+266/281=96%
Accuracy of do not send (expect low): 266/275=96.7%
Accuracy of do not send (expect high): 4/6=66.7%

Based on ME3 ONLY:

**Oncotype Score**	**Do not send-expect high**	**Do not send-expect low**	**Send**	**Grand Total**
>25	5	11	20	36
25 or less	1	286	49	336
**Grand Total**	**6**	**297**	**69**	**372**

%age classified as do not send: 6+297/372=81.5%
%age classified as send: 69/372=18.5%

**Oncotype Score**	**Do not send-expect high**	**Do not send-expect low**	**Grand Total**
>25	5	11	16
25 or less	1	286	287
**Grand Total**	**6**	**297**	**303**

Accuracy of do not send: 5+286/303=96%
Accuracy of do not send (expect low): 286/297=96.3%
Accuracy of do not send (expect high): 5/6=83.3%

Based on ME-mean:

**Oncotype Score**	**Do not send-expect high**	**Do not send-expect low**	**Send**	**Grand Total**
>25	5	9	22	36
25 or less		274	62	336
**Grand Total**	**5**	**283**	**84**	**372**

%age classified as do not send: 5+283/372=77.4%
%age classified as send: 84/372=22.6%

**Oncotype Score**	**Do not send-expect high**	**Do not send-expect low**	**Grand Total**
>25	5	9	14
25 or less		274	274
**Grand Total**	**5**	**283**	**288**

Accuracy of do not send: 5+274/288=96.9%
Accuracy of do not send (expect low): 274/283=96.8%
Accuracy of do not send (expect high): 5/5=100%
